# A Novel Benzo[f][1,7]Naphthyridine Produced by *Streptomyces Albogriseolus* from Mangrove Sediments

**DOI:** 10.3390/molecules15129298

**Published:** 2010-12-15

**Authors:** Xiao-Ling Li, Min-Juan Xu, Yi-Lei Zhao, Jun Xu

**Affiliations:** 1 Key Laboratory of MOE for Microbial metabolism and School of Life Science & Biotechnology, State Key Laboratory of Ocean Engineering, Shanghai JiaoTong University, Shanghai 200240, China; 2 Key Laboratory of Systems Biomedicine, Shanghai Center for Systems Biomedicine, Shanghai Jiao Tong University, Shanghai 200240, China; 3 Key Laboratory of Marine Biogenetic Resources, the Third Institute of Oceanography SOA, Xiamen, Fujian 361005, China

**Keywords:** *Streptomyces albogriseolus*, mangrove, benzo[f][1,7]naphthyridine

## Abstract

Mangrove *Streptomyces* represent a rich source of novel bioactive compounds in medicinal research. A novel alkaloid, named 1-*N*-methyl-3-methylamino-[*N*-butanoic acid-3′-(9′-methyl-8′-propen-7′-one)-amide]-benzo[f][1,7]naphthyridine-2-one (**1**) was isolated from *Streptomyces albogriseolus* originating from mangrove sediments. The structure of compound **1 **was elucidated by extensive spectroscopic data analyses and verified by the ^13^C-NMR calculation at the B3LYP/6-311+G(2d,p) level of theory.

## 1. Introduction

**Figure 1:**
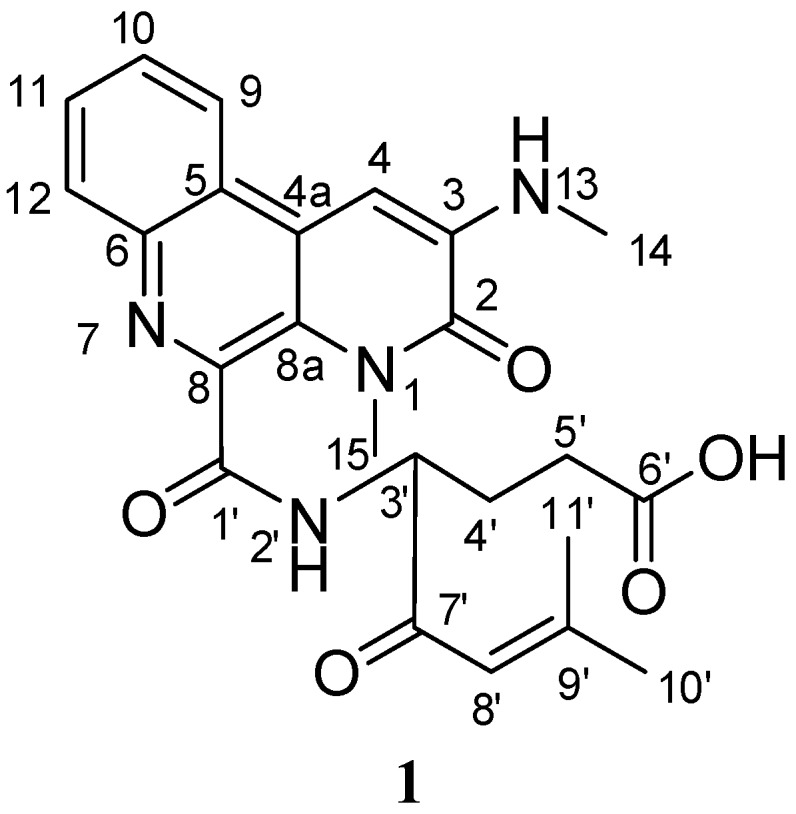
Novel benzonaphthyridine alkaloid **1** isolated from *S. albogriseolus.*

Mangrove sediments, which consisting of rich organic matter and salts represent some of the most complex microbial habitats on Earth. Culture independent molecular analysis has been intensively used to detect bacterial communities in mangrove sediments. At least eight different bacterial phyla including actinobacteria were detected in 16 S rRNA gene libraries constructed using a mangrove sediment of Sundarban, India [1]. Groups of actinobacteria strains were isolated from sediments collected from the national mangrove reserve in the Fujian Province of China and at least two novel *Streptomyces* species, *S. xiamenensis* [2] and *S. avicenniae* [3], were identified. The immense and untapped microbial biodiversity in the mangrove ecosystem is a promising resource for natural product studies. The actinobacterial strains that are subjected to the periodical changes in environmental factors seen in mangrove forests, such as salinity and tidal gradients, are believed to be effective selectors for metabolic pathway adaptations that could generate unique metabolites. Previous investigation on secondary metabolites from mangrove endophytes provided a number of new bioactive compounds [4,5,6,7,8]. *Streptomyces albogriseolus* is well-known for producing novel secondary metabolites and possessing good bioactivity. For instance, echinosporins isolated from marine-derived *S. albogriseolus* were found to be cell cycle inhibitors, apoptosis inducers and potential anti-tumor agents [9]. Antibiotics, namely cephamycin C, toyocamycin and neomycin complex, were also produced by the strain [10,11]. Thienodolin with its unique chloro-substituted structure was used as a plant growth-regulating substance [12]. A mangrove-derived *S. albogriseolus* was isolated from the sediments and its potential pharmaceutical applications were surveyed both genetically and chemically. This strain produced a novel benzonaphthyridine alkaloid (see Figure 1). In this paper, we report the structural elucidation of a new alkaloid identified as 1-*N*-methyl-3-methylamino-[*N*-butanoic acid-3′-(9′-methyl-8′-propen-7′-one)-amide]-benzo[f][1,7]naphthyridine-2-one (**1**) by extensive spectroscopic data analyses and the ^13^C-NMR calculations at the B3LYP/6-311+G(2d,p) level of theory.

## 2. Results and Discussion

**Figure 2:**
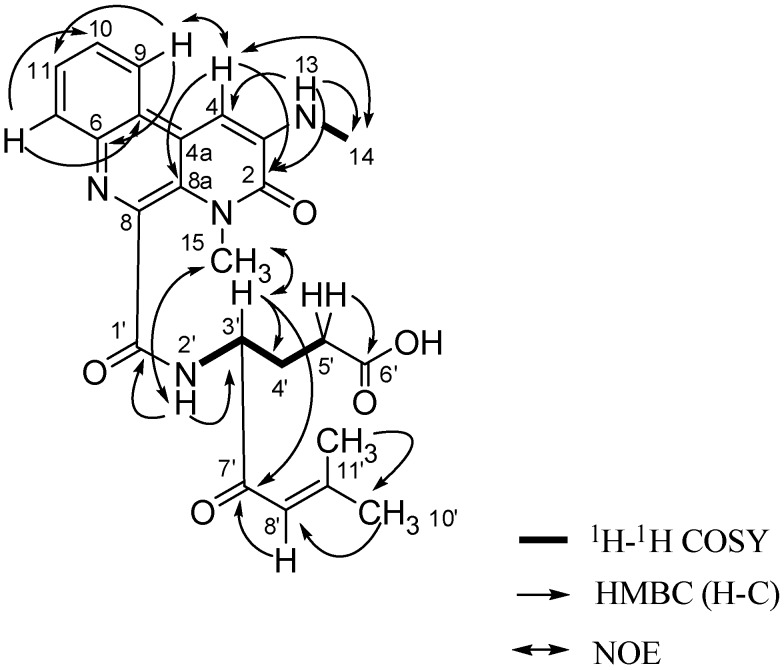
Key COSY, HMBC and NOE correlations of compound **1**.

Compound **1 **was isolated as an yellow amorphous powder and its molecular formula was determined to be C_24_H_26_N_4_O_5 _on the basis of HRESIMS analysis (451.2043 [M + H]^+^, calcd. for C_24_H_27_N_4_O_5_, *m*/*z* 451.1981), requiring fourteen degrees of unsaturation. The special bright red color of **1** on TLC after application of Dragendorff’s reagent is one of the obvious features of alkaloids, which was in accordance with the HRESIMS result. The UV maxima observed at 250, 380, 525 nm indicated a highly conjugated system. The IR absorptions at 3,427, 1,631, 1,609, 1,562 cm^−1^ suggested the presence of hydroxyl, amide carbonyl and aromatic groups, respectively. In the ^1^H-NMR spectrum, a four-proton spin system with signals at δ_H_ 8.63 (1H, d, *J* = 8.3 Hz), 7.68 (1H, dd, *J* = 8.3, 1.1 Hz),7.72 (1H, dd, *J* = 8.4, 1.1 Hz), and 7.98 (1H, d, *J* = 8.4 Hz), as well as a singlet aromatic proton at δ_H_7.28 (1H, s) were detected (See Table 1). Carbon signals of the ^13^C-NMR spectrum consisted of seventeen aromatic carbons, including six carbonyl groups (see Table 1) which supported the presence of a benzonaphthyridine moiety. The HMBC correlations from H-9 (8.63, d, *J* = 8.3 Hz) to C-6 (142.0, s), C-11 (128.6, d) and from H-12 (7.98, d, *J* = 8.4 Hz) to C-5 (123.2, s), C-10 (127.5, d) suggested the benzyl ring was connected to the naphthyridine ring (see Figure 2). HMBC correlations observed from H-4 (7.28, s) to C-2 (157.9, s), C-4a (123.0, s) and C-8a (125.8, s) indicated that the carbonyl group is placed on C-2. Based on the HMBC correlations from NH-13 (6.92, q, *J* = 5.1 Hz) to C-2 (157.9, s),C-4 (95.8, d) and CH_3_-14 (29.9 q), and the NOE interaction between H_3_-14 and H-4, the methylamino-group was positioned at C-3 (see Figure 2). Key NOE correlation between H-9 and H-4 confirmed the benzo[f][1,7]naphthyridine portion. In the aliphatic region, ^1^H-^1^H COSY spectrum revealed the signals at δ_H_ 4.57 (1H, ddd, H-3′), 1.89 (1H, dddd; 2.10, 1H, m, H_2_-4′) and 2.39 (2H, m, H_2_-5′) were adjacent to each other. HMBC correlation between H_2_-5′ and the carboxyl carbon C-6′ (δ_C_ 174.0) coupled with the presence of an exchangeable proton at (δ_H_ 12.23), indicated a carboxylic acid group on C-5′. A modified isoprenyl group consisting of an olefinic proton at δ_H_ 6.46 (H-8′, s) correlated to C-7′ (198.4, s), C-9′ (157.8, s) in the HMBC spectrum, ending the two methyls at δ_H_ 1.94 (H_3_-10′, s) and 2.16 (H_3_-11′, s), is attached to the C-3′ position of this aliphatic chain as indicated by the HMBC correlation between H-3′ to C-7′. The NOE interaction between NH-2′ (9.24, d, *J* = 7.4 Hz), H-3′ and H_3_-15 (3.71, s) suggested the aliphatic region and benzonaphthyridine portion was connected by the amide functional group (see Figure 2). 

**Table 1:** ^1^H- and ^13^C-NMR data of compound **1**^a^.

Position	1	
	δ_H_	δ_C_
1	--	--
2	--	157.9 s
3	--	142.2 s
4	7.28 s	95.8 d
4a	--	123.0 s
5	--	123.2 s
6	--	142.0 s
7	--	--
8	--	145.3 s
8a	--	125.8 s
9	8.63 d (8.3)	123.4 d
10	7.68 dd (8.3, 1.1)	127.5 d
11	7.72 dd (8.4, 1.1)	128.6 d
12	7.98 d (8.4)	129.5 d
13-NH	6.92 q (5.1)	--
14	2.98 d (5.0)	29.9 q
15	3.71 s	34.9 q
1′	--	169.2 s
2′-NH	9.24 d (7.4)	--
3′	4.57 ddd (8.4, 7.3, 4.8)	58.8 d
4′	2.10 m1.89 dddd (14.6, 8.4, 8.4, 8.4)	25.9 t
5′	2.39 m	30.8 t
6′	--	174.0 s
7′	--	198.4 s
8′	6.46 s	121.5 d
9′	--	157.8 s
10′	1.94 s	21.1 q
11′	2.16 s	28.0 q
6′-OH	12.23 s	--

^a^ Measured in DMSO-*d_6_*, Chemical shifts (δ) in ppm.

The structure of **1** was further confirmed by interpretation of the fragmentation pattern in HPLC-ESI-MS/MS. Ions from the benzonaphthyridine portion (238 u) and the aliphatic region (212 u) were found, representing the cleavage of the bond between C-1′ and C-8. The fragmentation pathway of benzonaphthyridine was found to result in the loss of methyl group and carbonyl group, successively, corresponding to the 223 u and 195 u in mass spectra. The mass fragment of 169 u displayed the loss of amide group from the metastable aliphatic ion. Thus, compound **1 **was determined as 1-*N*-methyl-3-methylamino-[*N*-butanoic acid-3′-(9′-methyl-8′-propen-7′-one)-amide]benzo[f][1,7]naphthyridine-2-one (**1**).

Compound **1** possesses a fused tricyclic heteroaromatic system, which belongs to diazaphenathrene family. Considering such structural character, calculation of ^13^C-NMR shifts has been applied as a useful tool for the structural elucidation, e.g. in the example of samoquasine A [13]. Here calculations of the theoretical ^13^C-NMR spectrum were carried out with the Gaussian 09 software package [14], using a simplified model in which the aliphatic chain was replaced with aldehyde. Geometry optimizations and frequency calculations were performed at the B3LYP/6-31G(d) level of theory and the optimized structures were verified as true minima with no imaginary frequency [15]. ^13^C-NMR chemical shifts were estimated with the GIAO-based method [16] at the B3LYP/6-311 + G(2d,p), using a benzene reference of 128.5 ppm. The calculated results were in good agreement with the experimental carbon resonance assignments and an unambiguous assignment of the ^13^C-NMR chemical shifts was achieved by the comparison of the computed ^13^C-NMR data with the experimental data (see Table 2). The deviation was found to be less than 5.5 ppm, and averaged 2.3 ppm. 

**Table 2:** Comparison of the calculated *vs.* experimental carbon resonances for the benzo[f][1,7]naphthyridine part of **1**.

Carbon	δ_expt__._	δ_ cald._	Δ (δ_ cald._ − δ_expt._)
2	157.9 s	158.8	+0.9
3	142.2 s	141.5	+0.7
4	95.8 d	93.2	+2.4
4a	123.0 s	127.4	+4.4
5	123.2 s	123.9	+0.7
6	142.0s	146.1	+4.1
8	145.3 s	144.3	+1.0
8a	125.8 s	129.2	+3.4
9	123.4 d	120.7	+2.7
10	127.5 d	128.2	+0.7
11	128.6 d	127.7	+1.1
12	129.5 d	135.0	+5.5

The benzonaphthyridines are unique alkaloids with diverse bioactivities. The marine environment seems to be a rich source of such molecules. Aaptamines were isolated from a marine sponge *Aaptos* sp*.* [17,18,19], which exhibited different bioactivities, e.g. competitive antagonist of α-adrenoceptors in vascular smooth muscles [20], activator of p21 promoter in a p53-independent manner [21], antineoplastic [22], antivirus and anticancer properties [23], hence triggering major interest in its total synthesis [24]. Another type of 2,7-naphthyridine alkaloids, the lophocladines and subarine were isolated from the marine red alga *Lophocladia* sp. [25] and a Singaporean ascidian, respectively [26]. In this work, benzo[f][1,7]naphthyridine was identified as the first example of this class found among mangrove related microbial metabolites. A series of such type of alkaloids was further detected by Dragendorff’s reagent on TLC and by HPLC-MS technique. An analogue of **1** possessing the molecular formula of C_24_H_28_N_4_O_6 _on the basis of HRESIMS analysis (469.1911 [M + H]^+^, calcd. for C_24_H_29_N_4_O_6_, *m*/*z* 469.2087) and similar UV profile was found in trace amounts, so more derivatives of benzo[f][1,7]naphthyridine seem to be produced by the *S. albogriseolus*, but their low productivity in fermentation hindered further chemical and pharmaceutical investigations. 

Primary bioinformatics analysis of the draft genome sequence of the producer strain, *S. albogriseolus* has revealed the existence of at least two putative antibiotic biosynthetic gene clusters, including polyketide synthases and key components in phenazines biosynthesis in the genome (unpublished data). Resolving the biosynthesis pathway of benzonaphthyridine will probably clarify the real source of such compounds in the microorganism. Considering the novelty of structure and the minor amounts isolated, the genetic manipulation of the biosynthetic genes might offer us another opportunity for searching for bioactive novel leading compounds besides synthetic methods and for producing such compounds in large amounts for medicinal research.

## 3. Experimental

### 3.1. General

^1^H- and ^13^C-NMR spectra were recorded with Bruker DRX-500 and Avance III-600 NMR spectrometers with TMS as an internal standard. Chemical shifts (δ) are expressed in parts per million (ppm) and coupling constants (*J*) are reported in Hertz (Hz). The IR spectra were recorded with a Bruker EQUINOX 55 FT spectrometer. Optical rotations were recorded on a JASCO P-2000 polarimeter. HR-ESI-MS spectra were measured on a Waters Quattro Premier XE mass spectrometer (Waters Corp., Millford, MA, USA). ESI-MS spectra were measured on a Bio ESI-Q-TOF mass spectrometer (Bruker Daltonik GmbH). Chromatographic separation for HPLC-ESI-MS/MS was carried out on Agilent ZOR BAS SB-C18 (ODS, 1.8 μm, 2.1 × 50 mm). Mass spectrometry for HPLC-ESI-MS/MS was carried out on a Agilent1200 MS Q-TOF 6530. CD spectra were taken on a J-815 spectropolarimeter (JASCO, Gross-Umstadt, Germany) at room temperature. Column chromatography was performed with silica gel (200-300 mesh, Qingdao Marine Chemical, Inc., Qingdao, P. R. China), silica gel H (10-40 μm, Qingdao), Sephadex LH-20 (40-70 μm, Amersham Pharmacia Biotech AB, Uppsala, Sweden) and Lichroprep reversed-phase RP-18 silica gel (40-63 μm, Merck, Darmstadt, Germany). Preparative TLC (1.0-1.5 mm) was conducted with glass-pre-coated silica gel GF254 (Qingdao), and spots were visualized under UV light. The chemical reagents used for chromatography were purchased from Shanghai Chemical Works Co. Ltd. (Shanghai). Analytical HPLC was carried out on an Agilent XDB-C18 column (4.6 × 150 mm, 5 μm), flow 1 mL/min, UV detection (254 nm). Organic solvents for HPLC were analytical grade were purchased from Merck KGaA (Darmstadt, Germany).

### 3.2. Strain Isolation, Characterization, and Cultivation

The strain was isolated from the sediment collected in the national mangrove reserve in Fujian province of China, where *Avicennia marina* grows as the dominant species*.* The collected sediment was air dried and ground up. Soil samples (1 g) were mixed with sterile water (9 mL) and then diluted to 10^−6^. The diluted suspension (100 μL) was transferred to Gause’s synthetic agar [soluble starch 20.0 g/L, K_2_HPO_4_ 0.5 g/L, MgSO_4_·7H_2_O 0.5 g/L, NaCl 0.5 g/L, NaNO_3_ 1.0 g/L, FeSO_4_ 0.01 g/L, agar 2%, 25 μg/mL nalidixic acid for inhibiting the grow of bacteria, pH 7.2] then incubated at 28 °C for10-15 days. Purified single colonies were classified according to colony morphology. One isolated strain MGR072 was assigned as *Streptomyces albogriseolus* by 16S rRNA gene sequence analysis. The production culture was carried out in a 30 L of ISP3 media (20 g oats per 1 L media, 1.5% agar, pH 7.2-7.4). It was grown for 12 days at 28 ± 1 °C.

### 3.3. Extraction and Isolation

The production culture of *S. albogriseolus* was extracted at room temperature overnight with a solvent mixture of ethyl acetate/methanol/acetic acid (about 5 L, 80:15:5, v:v:v). The supernatant was filtered and the residue was then extracted twice more as described above. The supernatants were combined and concentrated under vacuum at 37 ^o^C in order to remove the organic phase. The remaining aqueous solution was partitioned four times with ethyl acetate (about 2 L, 1:1, v:v). The upper layer was collected to afford a crude extract (6 g) which was subjected to column chromatography on RP-18, eluted by mixture of methanol and H_2_O [gradient from methanol, 30:70 (v:v, 2 L), 50:50 (v:v, 2 L), 70:30 (v:v, 2 L) to H_2_O], to afford five fractions Fr. 1-5. Fr. 3 (178 mg) was collected and further purified by repeated Sephadex LH-20 column chromatography, eluted with methanol (700 mL × 1, 250 mL × 2) first and then seperated again by Sephadex LH-20, using acetone(250 mL × 1) as solvent system. Subfraction 3-2 (13 mg) was identified as the target fraction by TLC which then applied to column chromatography on silica gel, eluted with chloroform/methanol [gradient from 50:1 (v:v, 100 mL) to 30:1 (v:v, 60 mL)], to afford *1-N-methyl-3-methylamino-[N-butanoic acid-3′-(9′-methyl-8′-propen-7′-one)-amide]-benzo[f][1,7]naphthyridine-2-one* (**1**, 5 mg). The compound was analyzed by HPLC after separation. Compound **1** (～1 mg/mL) was detected at the retention time of 31.7 min by analytical HPLC (Agilent XDB-C18, 4.6 × 150 mm, 5 μm), with the following gradient: H_2_O (A) / MeOH (B): 0 min 10% B, 10 min 10% B, 40 min 100% B, at flow rate of1 mL/min. Yellow amorphous powder; ${\left[\alpha \right]}_{D}^{22}$: −72° (*c* 0.0017, MeOH); UV *λ_ max_* (MeOH) 250, 380, 525 nm; CD (MeOH): Δ*ε*_195_ +0.6, Δ*ε*_201_ −0.6, Δ*ε*_205_ +0.3, Δ*ε*_220_ +0.4, Δ*ε*_227_ +0.3, Δ*ε*_231_ −0.1, Δ*ε*_237_ +0.4, Δ*ε*_249_ 0.2; IR (KBr) *ν_max _*3,427, 2,927, 1,631, 1,609, 1,562, 1,415, 1,350, 1,257, 762, 615 cm^−1^; ^1^H- and ^13^C-NMR data, see Table 1; ESIMS *m*/*z* 451.2 [M + H]^+^; HRESIMS *m*/*z* 451.2043 [M + H]^+^, (calcd. for C_24_H_27_N_4_O_5_, *m*/*z* 451.1981), 449.1622 [M − H]^−^, (calcd. for C_24_H_25_N_4_O_5_, *m*/*z* 449.1825); HPLC-ESI-MS/MS *m*/*z* 473.1745 [M + Na]^+^, 923.3739 [2M + Na]^+^, 238.0986, 223.0740, 212.0804, 210.0839, 195.0789, 184.0596, 181.0752, 169.0764.

### 3.4. Computational Methods

Structure optimization was accomplished with the program package Gaussian 09 using the hybrid DFT functional B3LYP together with Pople’s 6-31(d) and 6-311 + G(2d,p) basis sets. 

## 4. Conclusions

A novel benzonaphthyridine alkaloid has been isolated from a mangrove-derived *S. albogriseolus*. The compound has been identified as [*N*-butanoic acid-3′-(9′-methyl-8′-propen-7′-one)-amide]-benzo[f][1,7]naphthyridine-2-one (**1**) by extensive spectroscopic data analyses and quantum chemical carbon resonance calculations. Mangrove *Streptomyces* represent a rich source for novel bioactive compounds in medicinal research.
